# Monoclonal gammopathies of clinical significance (MGCS): In pursuit of optimal treatment

**DOI:** 10.3389/fimmu.2022.1045002

**Published:** 2022-11-23

**Authors:** Artem Oganesyan, Andrew Gregory, Florent Malard, Nerses Ghahramanyan, Mohamad Mohty, Dickran Kazandjian, Arsène Mekinian, Yervand Hakobyan

**Affiliations:** ^1^ Department of Hematology and Transfusion Medicine, National Institute of Health, Yerevan, Armenia; ^2^ Department Of Adult Hematology, Hematology Center after Prof. R. Yeolyan, Yerevan, Armenia; ^3^ Wayne State University School of Medicine, Detroit, MI, United States; ^4^ Department of Clinical Hematology and Cellular Therapy, INSERM, Saint-Antoine Research Centre, Assistance Publique-Hôpitaux de Paris, Hôpital Saint Antoine, Paris, France; ^5^ Myeloma Program, Sylvester Comprehensive Cancer Center, University of Miami, Miami, FL, United States; ^6^ Department of Internal Medicine (DMU i3), Sorbonne University, Assistance Publique-Hôpitaux de Paris, Hôpital Saint Antoine, Paris, France; ^7^ French-Armenian Clinical Research Center, National Institute of Health, Yerevan, Armenia

**Keywords:** monoclonal gammopathy, monoclonal gammopathy of clinical significance, MGUS, immunotherapy, monoclonal gammopathy of undetermined significance, MGCS

## Abstract

Monoclonal gammopathy of clinical significance (MGCS) represents a new clinical entity referring to a myriad of pathological conditions associated with the monoclonal gammopathy of undetermined significance (MGUS). The establishment of MGCS expands our current understanding of the pathophysiology of a range of diseases, in which the M protein is often found. Aside from the kidney, the three main organ systems most affected by monoclonal gammopathy include the peripheral nervous system, skin, and eye. The optimal management of these MGUS-related conditions is not known yet due to the paucity of clinical data, the rarity of some syndromes, and limited awareness among healthcare professionals. Currently, two main treatment approaches exist. The first one resembles the now-established therapeutic strategy for monoclonal gammopathy of renal significance (MGRS), in which chemotherapy with anti-myeloma agents is used to target clonal lesion that is thought to be the culprit of the complex clinical presentation. The second approach includes various systemic immunomodulatory or immunosuppressive options, including intravenous immunoglobulins, corticosteroids, or biological agents. Although some conditions of the MGCS spectrum can be effectively managed with therapies aiming at the etiology or pathogenesis of the disease, evidence regarding other pathologies is severely limited to individual patient data from case reports or series. Future research should pursue filling the gap in knowledge and finding the optimal treatment for this novel clinical category.

## Introduction

Monoclonal gammopathy of undetermined significance (MGUS) is a precancerous clonal plasma or lymphoplasmacytic proliferative disorder, which is defined by an asymptomatic appearance of monoclonal immunoglobulin (called M protein) in the serum at a concentration of <3 g/dL as well as less than 10% of bone marrow infiltration with plasma cells (1). MGUS is one of the most common premalignant conditions affecting 1-3% of adults which may lead to multiple myeloma (MM) (2, 3). In the contrast, MM is characterized by malignant plasma cell proliferation that produces M protein (usually >3g/dL) with ≥10% of bone marrow infiltration and is often manifested by end-organ damage commonly known as CRAB criteria (hypercalcemia, renal insufficiency, anemia, bone lytic lesions) (1). On average, MGUS has an annual progression rate of 1-2% (4) and is more prevalent in males and Blacks with increasing incidence in older adults (5). MGUS is mostly sporadic, although genetic predisposition may also play a role (6). There are 3 main clinical subtypes based on the type of M protein present: non-IgM, IgM, and light-chain MGUS. With regards to the risk of transformation into MM, MGUS is categorized as low, intermediate or high risk based on M protein level, type of M protein and free light chain ratio (7). Patients who do not meet the criteria for MM and have no symptoms are usually not treated, but rather monitored every 2-3 years for low-risk MGUS and annually for intermediate and high-risk MGUS for possible disease progression and potential complications, such as fractures, thromboembolic disease, or secondary malignancies (8).

Even in the absence of MM, various types of organ damage in the context of MGUS have been observed, involving neurological, skin, blood, and eye diseases. Importantly, the spectrum of these pathologies may range from single-organ disorders to systemic diseases. This new clinical entity is called monoclonal gammopathy of clinical significance (MGCS). Diagnosis of this disease is complicated by non-specific and alternating symptoms, poor understanding of pathogenesis, as well as complex clinical presentations. Although several disease pathways have been proposed, including monoclonal immunoglobulin deposition in tissues, autoantibody activity of M-protein, cytokine activation, and complement alternate pathway activation; the mechanisms are widely unknown (9). Moreover, the optimal management of these patients is unclear and yet to be determined.

One of the well-discussed examples is monoclonal gammopathy of renal significance (MGRS), in which kidney damage (e.g., tubulopathy, glomerulopathy, glomerulonephritis) caused by M-protein deposits, involving light chains, heavy chains, or intact immunoglobulins, occurs in the absence of MM or lymphoproliferative disorder. Rigorous research has led to an improved understanding of the disease, its diagnosis and management, in which the treatment is rather directed at protein-producing clones and type of pathological injury and not at histopathological features (10). Currently, anti-clonal therapy against either B-cells or plasma cells with novel anti-myeloma regimens (i.e., proteasome inhibitors, monoclonal antibodies, alkylating agents, immunosuppressants) is known to be more effective compared to immunomodulatory treatment commonly used for autoimmune-related renal diseases (11).

**Figure 1:**
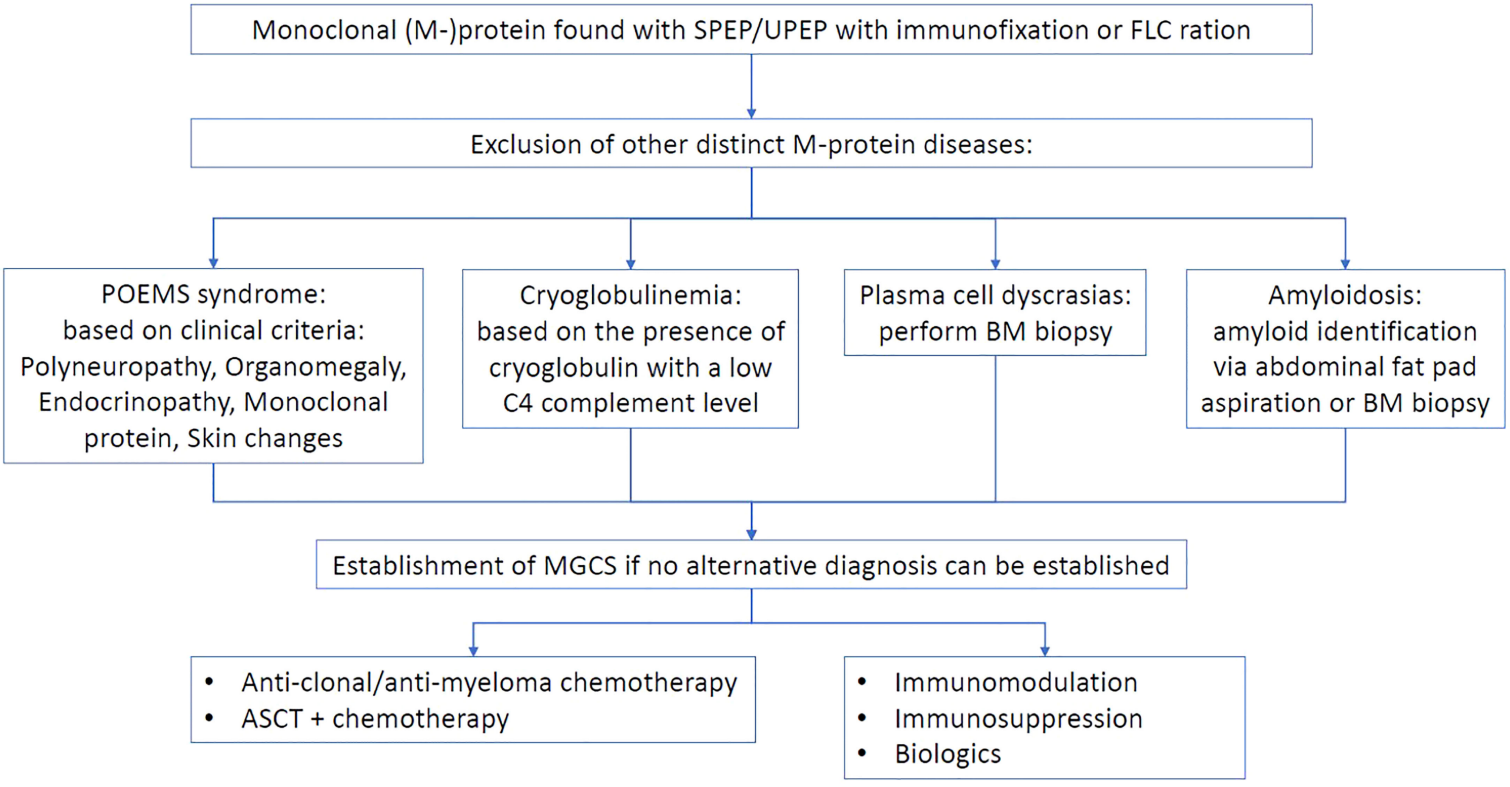
Proposed management approach to monoclonal gammopathy of clinical significance.

The biggest dilemma facing clinicians is the therapeutic target of MGCS. Two main approaches exist (**Figure 1**), one of which suggests an anti-paraproteinemic strategy that involves reduction or elimination one of which suggests an anti-paraproteinemic strategy that involves reduction or elimination of the aberrant clone-producing M protein. These various chemotherapeutic regimens aim to address the hematological aspect of MGCS suggesting that monoclonal gammopathy is the main etiological driver. The second approach focuses on immune modulation with common therapies, such as systemic corticosteroids, intravenous immunoglobulins (IVIG), or biologic agents, among others. This strategy supposes that immune dysfunction is the primary culprit of the complex disease. The choice between these approaches can be related to the possible pathogenesis of the disease (**Table 1**).

**Table 1:** Potential mechanisms of MGCS-related diseases.

Proposed mechanism	MGCS-related diseases
Immunoglobulin deposits	Crystal storing histiocytosis Crystalline keratopathy Maculopathy of monoclonal gammopathy
Autoantibodies	Acquired C1 inhibitor deficiency Acquired von Willebrand disease Xanthomatosis CANOMAD CIDP DADS-M
Uncertain	Clarkson’s disease TEMPI syndrome Neutrophilic dermatoses SLOMN Acquired cutis laxa Scleromyxedema Schnitzler syndrome

Fermand et al. (2018) (12).

CANOMAD, chronic ataxic neuropathy; ophthalmoplegia, IgM paraprotein, cold agglutinins, and disialosyl antibodies; CIDP, chronic inflammatory demyelinating polyneuropathy; DADS-M, distal acquired demyelinating symmetric neuropathy with monoclonal protein; MGCS, monoclonal gammopathy of clinical significance; SLOMN, sporadic late onset nemaline myopathy; TEMPI, telangiectasias, erythrocytosis and erythropoetininemia, monoclonal gammopathy, periphiric fluid accumulation, intrapulmonary shunting.

The present narrative literature review is based on an extensive literature search (described in the **Supplement**) and aims at describing the management of the various clinical disorders (aside from kidney pathologies) constituting the MGCS spectrum. Some clinical entities (e.g., POEMS or cryoglobulinemia) are not discussed in this article as they were addressed in several detailed reviews (13, 14).

## MGUS-associated peripheral nervous system involvement

Peripheral neuropathies have been associated with MGUS (most commonly of IgM origin) and were shown to be the most frequent indication for the diagnostic workup in this patient group, compromising almost a fifth of individuals with MGUS (15). The prevalence of M protein in peripheral neuropathies was estimated to range from 3 to 10% (16). Several mechanisms mediated by M protein activity have been proposed, including demyelination, binding to myelin-associated glycoprotein, as well as antiganglioside antibodies (15).

### Chronic inflammatory demyelinating polyneuropathy

Chronic inflammatory demyelinating polyneuropathy (CIDP) is a progressive and relapsing immune-mediated inflammation characterized by peripheral muscle weakness and sensory impairment (17). CIDP has multiple subtypes, one of which is associated with MGUS, in which monoclonal IgM antibodies are directed against myelin-associated glycoprotein (MAG) in around half of the patients leading to demyelination of distal large sensorimotor fibers (17). In a population-based study of 17,398 Minnesota residents (603 with MGUS and 16,793 controls), individuals with MGUS were shown to have a six-fold increased risk of CIDP compared to the general population (18). The typical picture of nerve biopsy includes the widening of the myelin lamellae as well as IgM and C3d deposits on myelin sheaths (18, 19). The primary treatment options for MG-associated CIPD involve plasmapheresis (19, 20), IVIG (21, 22), and steroids alone (23–25) or in combination with cyclophosphamide (19, 20, 25) (**Table 2**). Patients with slow progression and minimal symptoms may not require any intervention (19, 27). Interestingly, CIDP-MGUS is more responsive to plasma exchange (reaching on average 74%) compared to other types of CIDP, such as sensory, multifocal, or diabetes-associated (26). The cumulative efficacy of IVIG across studies is around 60% but ranges from 33% to 76%, likely attributable to other patient characteristics, such as disease severity or the origin of neuropathy (e.g., diabetes) (21–23, 27). Similarly, the success of plasma exchange (average response rate of 74%) and steroids (average response rate of 60%) in clinical improvements may also vary substantially from case to case (20, 38–40). Plasmapheresis can be effective in combination with chemotherapy or immunosuppression (20). The combination of cyclophosphamide with steroids gives a 55% response rate on average (19, 25). Adjunction of immunosuppressant (azathioprine, mycophenolate-mofetil, cyclosporin A) or immunomodulatory (lenalidomide) drugs may be warranted in treatment-resistant situations, but the data in this area is very limited (20, 24, 38–41). A case report on severe CIDP-MGUS showed a sustainable improvement with rituximab (42).

**Table 2:** Literature review on the management of MGUS-related peripheral nervous system disorders.

Author/Year	Patients number	MGUS type	Type of neurological involvement	Therapy	Outcome	MGUS response
Eureling 2002 (25)	25	IgG, IgM	CIDP-M	-Cyclophosphamide + prednisolone (18) -Dexamethasone (1) -IVIG (2) -Corticosteroids (2)	-Good clinical response (11/18) -Good response -No response -Good response at 1-3yrs of follow-up	N/A
Magy 2003 (24)	15	IgG, IgA	CIDP-M	Plasmapheresis, IVIG, corticosteroids	Sustained neurological improvement after few months	N/A
Gorson 1997 (26)	15	IgM-λ, IgM-κ, IgG-λ, IgG-κ	CIDP-M	-Plasmapheresis (12) -IVIG (6) -corticosteroids (2) Follow-up of >2 years	-Complete or partial response (10) -Complete or partial response (6) -Complete or partial response (2)	N/A
Tagawa 2000 (20)	8	IgM-κ, IgM-λ	CIDP-M	-Plasmapheresis +IFN-a (1)/ VMCP (1)/cyclosporin A (1)/cyclophosphamide (1) -IVIG (2) -Prednisolone (6), plasmapheresis (4)	-Complete or partial response -Mixed response -No response	N/A
Jann 2005 (22)	7	IgG, IgA	CIDP-M	-IVIG (7) Follow-up of 2 years	-Complete or partial response (5)	N/A
Kuitwaard 2015 (21)	21	IgG, IgM	CIDP-M	IVIG (21)	Complete or partial response (16)	N/A
Notermans 2000 (19)	20	IgM, IgG	CIDP-M	-Cyclophosphamide + prednisolone (15) -Azathioprine (3) -Plasmapheresis (2)	-Complete or partial response (7) -No response -Complete or partial response (1)	N/A
Vital 2000 (27)	18	IgM-λ, IgM-κ, IgG-λ, IgG-κ	CIDP-M	-IVIG (10) -Corticosteroids (11) -Chlorambucil (3) -Cyclophosphamide (2) -Azathioprine (3)	-Complete or partial response (5) -Complete or partial response (7) -No response -No response -No response	N/A
Le Cann 2020 (28)	41	IgM-κ, IgM-λ	CANOMAD	-IVIG (20) -Corticosteroids (11) -Chlorambucil (3) -Plasmapheresis (3) -CHOP (2) -Azathioprine (1) -Rituximab (1)	-Complete (4); partial (8); stabilization (6); progression (2) -Partial response (1); progression (10) -Partial response (1); progression (2) -Partial response (2); progression (1) -Partial response (1); progression (1) -Stabilization (1) -Partial response (1)	N/A
Garcia-Santibanez 2018 (29)	11	IgM-κ, IgM-λ, IgG-κ, IgG-λ	CANOMAD	-Rituximab (9) -IVIG (9) -Cyclophosphamide (4) -Corticosteroids (3), mycophenolate (1), plasmapheresis	-Complete response (8) -Partial response (5) -Partial response (2) -No response (4)	-Reduction (7), absent (4) at 5-26 years of follow-up
Notermans 1996 (30)	16	IgG-λ, IgG-κ IgM-λ, IgM-κ	Peripheral neuropathy	Cyclophosphamide + prednisolone (16) Follow-up of 3yrs	Partial or complete response (8) Stabilization (6)	Drop in BM infiltration; decrease in IgG/IgM
Mygland 2003 (23)	8	IgG IgM IgA	DADS-M CIDP-M	IVIG, prednisolone	DADS-M: No response; CIDP-M: Partial response in >80%	N/A
Katz 2000 (31)	8	IgM-κ IgM-λ	DADS-M (3), CIDP-M (5)	Prednisolone, IVIG, plasmapheresis oral cyclophosphamide	DADS-M: No response CIDP-M: Improvement in motor function with prednisolone & plasma exchange being the most effective	N/A
Chahin 2005 (32)	4	IgG-κ, IgG-λ	SLOMN	Prednisolone (+ cyclophosphamide or IVIG)	No response (3), stabile for 4.5 years (1)	N/A
Voermans 2014 (33)	8	IgG-κ, IgG-λ	SLOMN	AHCT (8)	Complete partial response lasting for 1-6 years	
Naddaf 2019 (34)	17	IgG-κ, IgG-λ	SLOMN	-IVIG -AHCT -Chemotherapy -Immunosuppressive therapy	-Partial response (years) -Partial response (5 years) -Partial response -No response (3 years)	N/A
Schnitzler 2017 (35)	26	IgG-κ, IgG-λ	SLOMN	-Immunosuppressive therapy (19) -IVIG (7) -AHCT (7) -Plasmapheresis (2)	-Complete or partial response (6) -Complete or partial response (3) -Complete or partial response (6) -No response	N/A
Monforte 2018 (36)	6	IgG-κ, IgG-λ	SLOMN	-Prednisolone + IVIG (3) (+ azathioprine (2), + bortezomib + melphalan (1))	-Complete or partial response in 5/6 (at 16-36 months)	-Not detectable -Raised steadily
Okhovat 2020 (37)	3	IgG-κ, IgG-λ	SLOMN	(Methyl) prednisolone + IVIG (3)	Complete or partial response (at 6 months)	N/A

AHCT, autologous hematopoietic cell transplantation; BM, bone marrow; CANOMAD, chronic ataxic neuropathy, ophthalmoplegia, IgM paraprotein, cold agglutinins, and disialosyl antibodies; CHOP, cyclophosphamide + doxorubicin + vincristine + prednisone; CIPD-M, chronic inflammatory demyelinating polyneuropathy with MGUS; DADS-M, Distal acquired demyelinating symmetric neuropathy with monoclonal protein; IVIG, intravenous immunoglobulin; N/A, not available; SLOMN, Sporadic late-onset nemaline myopathy; VMCP, vincristine + melphalan + cyclophosphamide + prednisolone.

### Distal acquired demyelinating symmetric neuropathy with monoclonal protein

A slightly distinct peripheral nervous system disorder associated with MGUS is distal, acquired, demyelinating, symmetric neuropathy with M protein (DADS-M), which occurs in older males affecting large sensory nerve fibers, resulting in sensory ataxia and diminished sensory response, and motor neurons with decreased motor conduction velocity and prolonged distal latencies (43). Muscles of the face, proximal limbs and trunk are usually intact (44). This syndrome is distinct from the classical DADS by the clinical picture and pathogenesis, which is recognized as a subtype of CIDP. Nonetheless, the treatment, which is largely similar to the one described for CIDP with comparably worse outcomes overall, is based primarily on the severity of neurological symptoms, rather than on the levels of IgM. Moreover, the detection of anti-MAG, which is present in about 50-70% of patients (44, 45), takes a decisive role in the choice of therapy as it is mostly resistant to standard treatment options (28, 41), such as IVIG, plasmapheresis, or systemic glucocorticoids (44, 45). Second-line treatment includes rituximab, lenalidomide, carfilzomib, or cyclophosphamide, the data on which is however limited (40).

### Chronic ataxic neuropathy, ophthalmoplegia, IgM paraprotein, cold agglutinins, and disialosyl antibodies

Another complex syndrome observed in patients with MGUS is chronic ataxic neuropathy, ophthalmoplegia, IgM paraprotein, cold agglutinins, and disialosyl antibodies (CANOMAD). The proposed pathophysiology of CANOMAD involves IgM-mediated autoantibodies against gangliosides with disialosyl groups affecting sensory, ocular, and bulbar nerves leading to gait problems, muscle weakness, and paresthesia (28, 29). The disease is progressive in its nature with possible chronic relapses (28). Several effective treatment options exist based on data from case reports and series, with the most supported being IVIG (46–48) and rituximab-based strategies (46, 47, 49, 50). A French multicenter retrospective study of 45 patients with CANOMAD found 53% and 52% clinical response with IVIG and rituximab, respectively, while immunosuppressants were not shown to be particularly beneficial (28). These findings were supported by an earlier series of 11 cases (29), although some case reports claimed the efficacy of steroids (51, 52).

### Sporadic late onset nemaline myopathy

Sporadic late-onset nemaline myopathy (SLOMN) is characterized by subacute and progressive muscular weakness, pain, and atrophy involving myofibers of the proximal limbs, neck, and face (36). Clinical presentation typically includes head drop, dysphagia, dysarthria, nemaline bodies in the cytoplasm, heart failure, and severe respiratory insufficiency leading to death within a few years of onset (32, 35, 36). It occurs in middle-aged men and women with more than half of patients having MGUS (34, 35). The exact mechanism and etiology of SLOMN are unclear. For the first-line treatment of SLOMN autologous hematopoietic cell therapy (AHCT) following high-dose melphalan should be considered, which demonstrated high rates of favorable response with hematologic and muscular improvements with over half of patients having positive long-term outcomes (33, 35, 37, 53). Chemotherapy directed against MM (lenalidomide with dexamethasone, rituximab with cyclophosphamide, or bortezomib with cyclophosphamide and dexamethasone) for patients not suitable for AHCT was shown to lead to some positive outcomes, such as disease stabilization and improved muscular performance (33, 53). IVIG is another alternative with comparably modest efficacy (35, 36, 53). Plasmapheresis is only partially effective, whereas steroids and other immunosuppressive therapies showed not promising outcomes for patients with SLOMN (30, 32, 36, 37, 54).

## MGUS-associated cutaneous involvement

**Figure 2:**
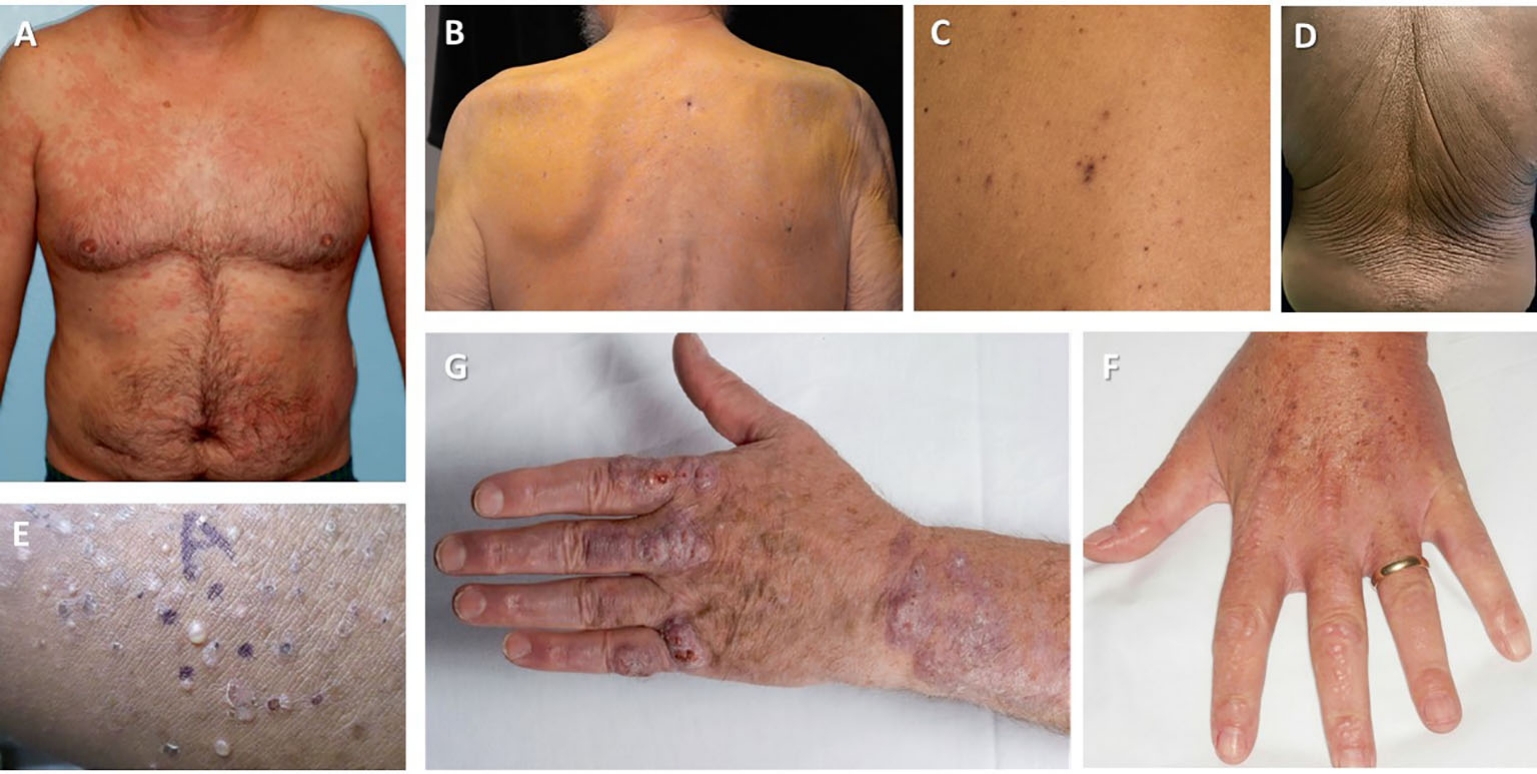
Cutaneous manifestations of MGCS. **(A)** Schnitzler syndrome (Image courtesy: Wilmas et al. 2018 (55)) **(B)** Nonhyperlipidemic xanthomatosis (Image courtesy: Cohen et al. 2015 (56)) **(C)** Telangiectasias in TEMPI syndrome (Image courtesy Khan et al., 2014 (57)) **(D)** Acquired cutis laxa (Image courtesy: Shalhout et al., 2010 (58)) **(E)** Subcorneal pustular dermatosis (Image courtesy: Young et al., 2021 (59)) **(G)** Necrobiotic xanthogranuloma (Image courtesy: Inthasotti et al. 2010 (60)) **(F)** Scleromyxedema (Image courtesy: Claveau et al. 2022 (61)).

Skin is one of the most commonly affected organs by a clonal proliferation of lymphocytes or plasma cells (**Figure 2**). Aside from Waldenström macroglobulinemia and amyloidosis (discussed elsewhere), which are the result of extravascular depositions of M-protein, a myriad of other cutaneous manifestations can be caused by vascular deposits, abnormal cytokine response, or pathological activity of immunoglobulins (62) (**Table 3**).

**Table 3:** Literature review of the management of MGUS-related skin disorders.

Author/Year	Patients number	MGUS type	Type of skin involvement	Type of therapy	Outcome	MGUS response
de Chambrun 2017 (63)	57	IgG-κ, IgG-λ	Clarkson disease	-IVIG (48) -Terbutaline (22) Median follow up of 5.1 years	-Complete/partial response -No response	-Lower IgG levels -No response -MM (5)
Kapoor 2010 (64)	19	IgG	Clarkson disease	-Methylxanthines (23), terbutaline (21), leukotriene inhibitor (10) -Zafirlukast + lisinopril (2) Median follow up of 4.9 years	-No response -Partial response	N/A
Wood 2009 (65)	17	κ, γ light chains	Necrobiotic xanthogranuloma	-Chemotherapy (2) -Chlorambucil + prednisolone (4) -Melphalan + prednisolone (1) -Dexamethasone (1) -Thalidomide + prednisolone (1) -Rituximab (2) -Intralesional corticosteroids + topical immunomodulators (2)	-Partial response (2) -Complete response (2) -Partial response (1) -Partial response -2 years of remission -Complete response (1) -No response	N/A
Higgins 2016 (66)	28	IgG-κ, IgG-λ, IgM-κ	Necrobiotic xanthogranuloma	-AHCT (3) -Chlorambucil ± corticosteroids (5) -FCR (1) -Melphalan + corticosteroids (3), VDD (2), antibiotics (3), cyclophosphamide + corticosteroids (4) -Corticosteroids (11) -Rituximab (6) -IVIG (4) -Thalidomide ± corticosteroids (11) -Lenalidomide ± corticosteroids (11) -Bortezomib ± corticosteroids (4) -BLD (1)	-Complete response (2) -Complete response (2) -Complete response (1) -No response -Complete response (4) -Complete response (1) -Complete response (2) -Complete response (4) -Complete response (7) -Complete response (1) -Complete response (1)	N/A
Szalat 2011 (67)	4	IgG-κ, IgG-λ, IgM-κ	Necrobiotic xanthogranuloma	-Thalidomide + bortezomib -Corticosteroids + chlorambucil -Chlorambucil + rituximab + fludarabine + cyclophosphamide + thalidomide + dexamethasone	-Partial response (1) -Partial response (2) -Complete response (1)	N/A
Donato 2006 (68)	7	IgG-κ, IgG-λ	Scleromyxedema	AHCT	Complete (5), partial response (2)	N/A
Kreuter 2005 (69)	4	IgG-κ, IgG-λ	Scleromyxedema	-IVIG (4), cyclophosphamide (1) -Dexamethasone (3) -Bortezomib (1) -Phototherapy, acitretin, methotrexate, thalidomide	-Partial response, recurred -Complete response, recurred -Complete response (1) -No response	N/A
Mahevas 2020 (70)	31	IgG-κ, IgG-λ	Scleromyxedema	-IVIG (21) -IVIG + corticosteroids (10) -IVIG + lenalidomide (1) -IVIG + thalidomide (1) -Lenalidomide (3) -Thalidomide (3) -Acitretin (2) -Corticosteroids (3) -Melphalan + dexamethasone (2) -Methotrexate (1)	-Complete or partial response -Complete or partial response -Complete response -Partial response -Weak response -Weak response -No response -Complete or no response -Weak response -No response	-Complete response with IVIG
Rongioletti 2013 (71)	25	IgG-κ, IgG-λ	Scleromyxedema	-IVIG (11) -IVIG + lenalidomide (1) -IVIG + corticosteroids (1) -Thalidomide (1) -Acitretin (1) -Mycophenolate (1) -Prednisolone + hydroxychloroquine (1) -Prednisolone + thalidomide (1) -Corticosteroids (2) -Mycophenolate (1) -Other therapies	-Complete/partial response (3/6) -Complete response (1) -Complete response (1) -Partial response (1) -Partial response (1) -Partial response (1) -Partial response (1) -Partial response (1) -No response (2) -No response (1) -No response	N/A
Terpos 2012 (72)	13	IgM-κ, IgM-λ, IgG-κ, IgG-λ	Schnitzler syndrome	-Perfloxacin (8) -Anakinra (7)	-Complete response (5) -Complete response (7)	N/A
Sokumbi 2012 (73)	20	IgM-κ, IgM-λ, IgG-κ, IgG-λ	Schnitzler syndrome	-Corticosteroids (13) -Rituximab (3), -Cyclophosphamide (1) -Anakinra (2)	-Partial response (11) -Partial response (2) -Partial response (1) -Partial response (1)	Malignant transformation (9)
Gusdorf 2017 (74)	25	IgM, IgG	Schnitzler syndrome	-Anakinra (29)	-Complete response (23)	N/A
Neel 2014 (75)	42	IgM-κ	Schnitzler syndrome	-Anakinra or canakinumab	-Complete response (29) at 36 months of follow up	N/A

AHCT, autologous hematopoietic cell transplantation; BLD, bortezomib + lenalidomide + dexamethasone; FCR, fludarabine + cyclophosphamide + rituximab; FLD, fludarabine; MM, multiple myeloma; N/A, not available; R-CHOP, cyclophosphamide, hydroxydaunorubicin, vincristine, prednisolone; VDD, vincristine + doxorubicin + dexamethasone.

### Schnitzler syndrome

Schnitzler syndrome is a rare systemic late onset autoinflammatory disease characterized by periodic fever, urticarial rash (neutrophilic urticarial dermatosis), bone pain with osteosclerotic changes, myalgia, lymphadenopathy, and arthralgia, as well as immunoproliferative disorders, such as B-cell lymphoma or MGUS (primarily IgM with kappa component). The disease mainly affects middle-aged adults of all ethnic groups and both sexes (76). Complications include lymphoproliferative disorder and AA amyloidosis, if left treated (76, 77). Schnitzler syndrome is thought to be a result of abnormal activation of the innate immune system with aberrant functioning of cytokines (77). Data from individual cases and clinical trials indicate that the most effective treatment option to date is anakinra (100 mg), the IL-1 receptor antagonist, which targets the key pathogenic mechanism of this disease and leads to complete remission in over 80% of patients (72, 74, 75, 78–82). Other therapies blocking IL-1 include rilonacept (IL-1 inhibitor) and canakinumab (IL-1 beta inhibitor), both of which demonstrated substantial clinical efficacy (83, 84). Second-line therapies, used previously before the introduction of anti-IL-1 treatment, include systemic glucocorticoids, NSAIDs, antihistamines, immunosuppressants, biologics, antimetabolites, showing limited efficacy and unfavorable safety profile overall (72, 73, 80, 81, 85–87). Emerging data also suggests promising use of Bruton tyrosine kinase (BTK) inhibitors (e.g., ibrutinib) as a mode of anti-clonal therapy (88).

### Scleromyxedema

Scleromyxedema is a rare cutaneous mucinosis with some systemic manifestations in association with MG. It usually occurs in middle-aged adults and is characterized by a generalized papular rash with sclerosis, which is the result of fibrosis and mucin deposition (70, 71). Multiorgan involvement (e.g., cardiac, digestive, lung, kidney, musculoskeletal and nervous systems) is the main cause of high morbidity and mortality (71). The etiology and pathogenesis of scleromyxedema are not fully clear but include fibroblast proliferation which might be stimulated by cytokine dysregulation and paraproteins (89). MGUS mostly involves IgG with lambda light chain (90). Based on case series and observational studies, the preferred treatment usually includes IVIG with systemic glucocorticoids, and immunomodulatory drugs (thalidomide or lenalidomide) being a second-line choice. High-dose IVIG (2g per kg given over 5 consecutive days every 4-6 weeks) has been shown to result in clinical remission of the disease for non-severe forms of scleromyxedema (i.e., without cardiac or CNS involvement) (71). The majority of the patients reach at least partial response after 4-6 cycles of this treatment, although improvements might be already visible after just the first two cycles as well (70). Remission may last from a few months to several years; therefore, maintenance therapy (every six to twelve weeks) is often warranted. For severe cases (i.e., refractory to high-dose IVIg or with cardiac and CNS involvement), anti-plasma cell therapies should be advocated.

Alternative therapies include thalidomide or lenalidomide, which are combined with IVIG or used in patients that cannot receive the latter one. Thalidomide. given at a dose of 50-100mg/day and further increased to 200-400mg/day, was shown to lead to improvements in skin lesions and paraprotein concentrations, as well as amelioration of some clinical symptoms within several months to a few years (91–93). Thalidomide adjunction to IVIG may also potentiate the therapeutic effects in complex cases (94). The limitations of the treatment include side effects (peripheral neuropathy) and the length of therapy required until clinical results are seen. Lenalidomide (10-25mg/day for days 1–21 of a 28-day cycle) has a better safety profile but has been tested only in combination with dexamethasone and IVIG (70, 95).

Systemic glucocorticoids (prednisone at 0.5-1 mg/kg per day, prednisolone at 0.3-0.5 mg/kg per day, or oral dexamethasone at 40 mg/day) are another option when the initial therapy has failed. They can be applied either alone or in combination with IVG or thalidomide. The mixed efficacy is based on the data from the case series showing regression of skin manifestations (69), although treatment failure has also been reported (70, 71).

Bortezomib (1.3 mg/m^2^, on days 1, 4, 8, and 11 every 21 days) in combination with 40 mg dexamethasone has been reported in severe and refractory cases with some success (96, 97) as well as in dermato-neuro syndrome, which is an acute and potentially fatal neurological complication (98). Successful addition of thalidomide to this regimen was also described (99).

Melphalan, a chemotherapeutic agent, has been previously widely used for scleromyxedema but was later abandoned due to severe adverse events, such as sepsis or secondary hematological malignancy (100). Currently, it may be combined with other therapies, such as IVIG, AHCT, or glucocorticoids (68, 100, 101).

Several case reports described high dose melphalan followed by AHCT in selective patients to result in partial remission of systemic manifestations of scleromyxedema (68, 102, 103). Similarly, plasmapheresis has been described to be used in severe and acute cases of scleromyxedema with some efficacy (70).

A myriad of other therapeutic options tried for the treatment of scleromyxedema included retinoids (acitretin and oral isotretinoin) (71, 104), immunosuppressants (mycophenolate mofetil, cyclosporine) (105, 106), and biologics (TNF-alpha inhibitors, interferon-alfa) (107, 108), and chemotherapeutics (methotrexate, cyclophosphamide) (109, 110). The efficacy of these options needs to be further investigated.

### Necrobiotic xanthogranuloma

Necrobiotic xanthogranuloma (NXG) is another idiopathic cutaneous pathology associated with paraproteinemia observed in older adults with a mean age of 62 years (111). Classically, it is described as a non–Langerhans cell histiocytosis manifested with papules, plaques, or nodules of various colors most commonly on the periorbital skin, although other regions of the body can also be affected (66). Systemic lesions with ocular, gastrointestinal, cardiac, and respiratory involvement may also occur (65). It has been estimated that 82% of the patients with NXG present with MG, with IgG-kappa being the most common subtype followed by IgG-lambda, IgG-kappa, IgA, and IgM (111).

The optimal treatment of NXG is still unclear providing undefined pathogenesis of the disease. Chemotherapy is reasonable for patients with underlying malignancy (e.g., multiple myeloma or chronic lymphocytic leukemia). Alkylating agents, such as chlorambucil (2-4mg/day) or melphalan (10mg), alone or in combination with other systemic therapies, were shown to result in cutaneous lesion improvements and lesser normalization in paraproteinemia in retrospective observational studies and case reports (65, 67, 112–114). Severe adverse events however limit the applicability of both drugs. Alternatively, oral cyclophosphamide (1 mg/kg per day for six months) can be used alone or in combination with steroids (112, 115, 116). Similarly, bortezomib alone or combined with steroids and/or lenalidomide/thalidomide is another choice for patients for whom chemotherapy is an option as it may lead to improved skin disease (66, 67). Successful application of IVIG inducing a complete or partial clinical response was described in several reports (66, 111, 117). Systemic glucocorticoids (pulsed dexamethasone or prednisone) were associated with symptomatic regression of NXG in a series of cases (65, 118). Some limited data also exist on benefits from the treatment with thalidomide and lenalidomide, including remission of skin lesions and decrease in gammopathy (65, 119, 120). Plasmapheresis or AHCT should be reserved for refractory cases (65, 121). Patients with more localized scenarios may benefit from topical or intralesional preparations, such as immunomodulators or steroids, as well as ultraviolet or radiotherapy (122–125). Surgery is an important component of the management of these patients both from cosmetic and functional point of view (126, 127). Other potential therapy options may include dapsone (128), antimalarials (111), and biologics (125, 129, 130).

### Hyperlipidemic and nonhyperlipidemic xanthomatosis

Xanthomatosis is a skin manifestation (cholesterol depositions) of a disturbance in lipid metabolism with or without hyperlipidemia. The condition is often associated with MGUS of IgG lambda or IgG kappa chains (131–134). Data on the treatment of xanthomatosis is lacking, but one report showed regression of xanthomas with probucol combined with topical steroid and oral antihistamines (132). However, others failed to demonstrate any benefits with cholestyramine, gemfibrozil (131), or steroids (67, 124). Chemotherapy for underlying hematological malignancy led to positive outcomes in some patients but did not show results in others (131, 133).

### TEMPI syndrome

TEMPI syndrome (Telangiectasias, Erythrocytosis and Erythropoetininemia, Monoclonal Gammopathy, Periphiric fluid accumulation, Intrapulmonary shunting) is a very rare, acquired disease manifested in middle-aged men and women across all ethnicities around the world (135, 136). The disorder has no definitive identified genetic component and is not fully clear in its pathogenesis (although the role of macrophage migration inhibitory factor (MIF) was suggested in the development of disease) (137), and MGUS, seen in all reported patients, is not restricted to any specific type, unlike in other conditions described here (135, 136). As of 2022, there have been a little more than 30 cases reported worldwide (135–147). Therefore, the treatment options are based on individual data only. To date, bortezomib is the most frequently tested treatment revealing mostly positive outcomes in several reports (135, 138–142). Treatment with daratumumab (anti-CD38 monoclonal antibody) was shown to elicit a complete symptomatic remission in two patients (143), but did not help in the management of another individual (144). AHCT was also described and resulted in complete hematological remission in one patient but was unsuccessful in two other cases (138, 145, 146). Similarly, lenalidomide was also attempted with ambiguous clinical results (143, 147).

### Acquired cutis laxa

Acquired cutis laxa is a rare form of connective tissue disorder manifested by loose and inelastic skin due to the degradation of cutaneous elastic fibers. Patients of different ages usually present with “premature aging skin”, which has multiple wrinkled lesions (148). It has been often associated with systemic and cutaneous inflammatory conditions, drug exposure, as well as hematological malignancies and MG, which primarily contains IgG lambda or kappa light chains (58, 148–152). Treatment of acquired cutis laxa is targeted at associated hematological or systemic diseases as no specific options (aside from reconstructive surgery or laser tightening) exist for that condition (148, 149). It is also believed that the management of related disorders will lead to dermatological improvement (58, 150–152); however, this hypothesis has not been proven yet.

### Neutrophilic dermatosis

Neutrophilic dermatosis is a diverse group of skin disorders characterized by severe infiltrations involving different cutaneous layers and manifests as ulcerations, pustules, ulcers, or nodules (153–155). Although neutrophilic dermatosis is often associated with inflammatory conditions and extracutaneous involvement, the disease itself is not mediated by infections or vasculitis (154). The exact mechanisms of pathologies are not well understood, and histopathology varies from type to type (154, 155). Despite evolving evidence suggesting the role of myeloid dysfunction in neutrophilic dermatosis, some forms of the disease have been associated with MGUS, including pyoderma gangrenosum, erythema elevatum diutinum, subcorneal pustular dermatosis, Sweet’s syndrome, and neutrophilic urticarial dermatosis (153–156). Treatment varies depending on the type of neutrophilic dermatosis. MGUS mostly relates to IgA and rare IgG with kappa or lambda bounds (153–168).

For *pyoderma gangrenosum*, which is characterized by bullous or pustular painful ulcers, wound management is the key treatment component with application of topical corticosteroids and local calcineurin inhibitors for localized disease (162). Systemic glucocorticoids or cyclosporine are usually considered for more advanced cases (163, 164). In patients with concomitant MG, systemic glucocorticoids and dapsone (with or without minocycline) have been the most commonly used options with mixed results (153, 158). Bortezomib–dexamethasone regimen led to resolution of lesions in one report (161). Other potential options described in the literature include colchicine, splenectomy, thalidomide, cyclophosphamide, clofazimine, methotrexate, IVIG, and azathioprine, the efficacy of which is yet to be determined (153).

*Subcorneal pustular dermatosis* (also known as Sneddon-Wilkinson disease) is defined by annular flaccid pustules localized to the axial and inguinal regions (165). Standard therapy involves dapsone, systemic glucocorticoids, or phototherapy (166). Acitretin (25 mg/day or higher) was described in several patients with concurrent gammopathy to lead to the resolution of lesions (153, 157, 159, 163). The potential use of biologic agents (infliximab, etanercept, and adalimumab) was also described in another case report (160).

*Sweet syndrome*, also known as acute febrile neutrophilic dermatosis, is a systemic inflammatory condition characterized by widespread erythematous papules or plaques with neutrophilic infiltrates as well as arthritis, fever, and neutrophilia. It is classified as idiopathic, drug-induced or malignancy-associated (167). Traditionally, systemic, topical, or intralesional glucocorticoids are the first-line treatment choice (168). Alternative options include colchicine, dapsone (168). The presence of monoclonal gammopathy seems to not alter the treatment approach in these patients (153).

*Neutrophilic urticarial dermatosis* is a chronic and recurrent condition with erythematous macules and plaques on extremities and trunk usually resolving in 24-48 hours after eruptions without any residual lesions (169). Aside from being a pathologic hallmark of Schnitzler syndrome, it is commonly associated with adult-onset Still’s disease, lupus erythematosus, and cryopyrin-associated periodic syndromes (169). Dapsone, colchicine, topical steroids, and anakinra are linked to clinical improvements (153, 155, 170).

### Clarkson disease (systemic capillary leak syndrome)

Clarkson disease or systemic capillary leak syndrome, first described in 1960, is characterized by sporadic and recurrent episodes of hypovolemic shock and anasarca, which are caused by widespread leakage of plasma and proteins into the extravascular compartment of various tissues and subsequent hypoalbuminemia and hemoconcentration (171, 172). Complications include pulmonary edema, compartment syndrome, and ischemic damage of organs (64, 173). MGUS seen in a majority of patients is IgG kappa or lambda light chains (63, 64). Despite the potential pathologic role of elevated levels of vascular permeability factors (174), the exact etiology is unknown, and the severity of the clinical manifestations varies from case to case (63). Judicious fluid resuscitation and hemodynamic support with the aim to restore the perfusion but at the same time avoid potential complications (e.g., pulmonary edema and compartment syndrome) are key for the management of acute episodes of hypovolemic shock (175, 176). A number of reports described successful application of high-dose IVIG (2g/kg) as a prophylactic measure and long-term treatment leading to a decrease in severity and frequency of attacks (63, 175). Terbutaline with aminophylline or theophylline was reported in several patients with variable efficacy for the prevention of subsequent episodes (173, 175–178). In a study of 69 patients with Clarkson disease, treatment with IVIG was associated with lower mortality and fewer recurrence rate compared to terbutaline in a median follow-up of 5 years (63). Limited data also exist regarding treatment with bevacizumab, infliximab, verapamil, thalidomide, leukotriene inhibitors, and glucocorticoids (64, 176, 179–181).

## MGUS-associated ocular involvement

**Figure 3:**
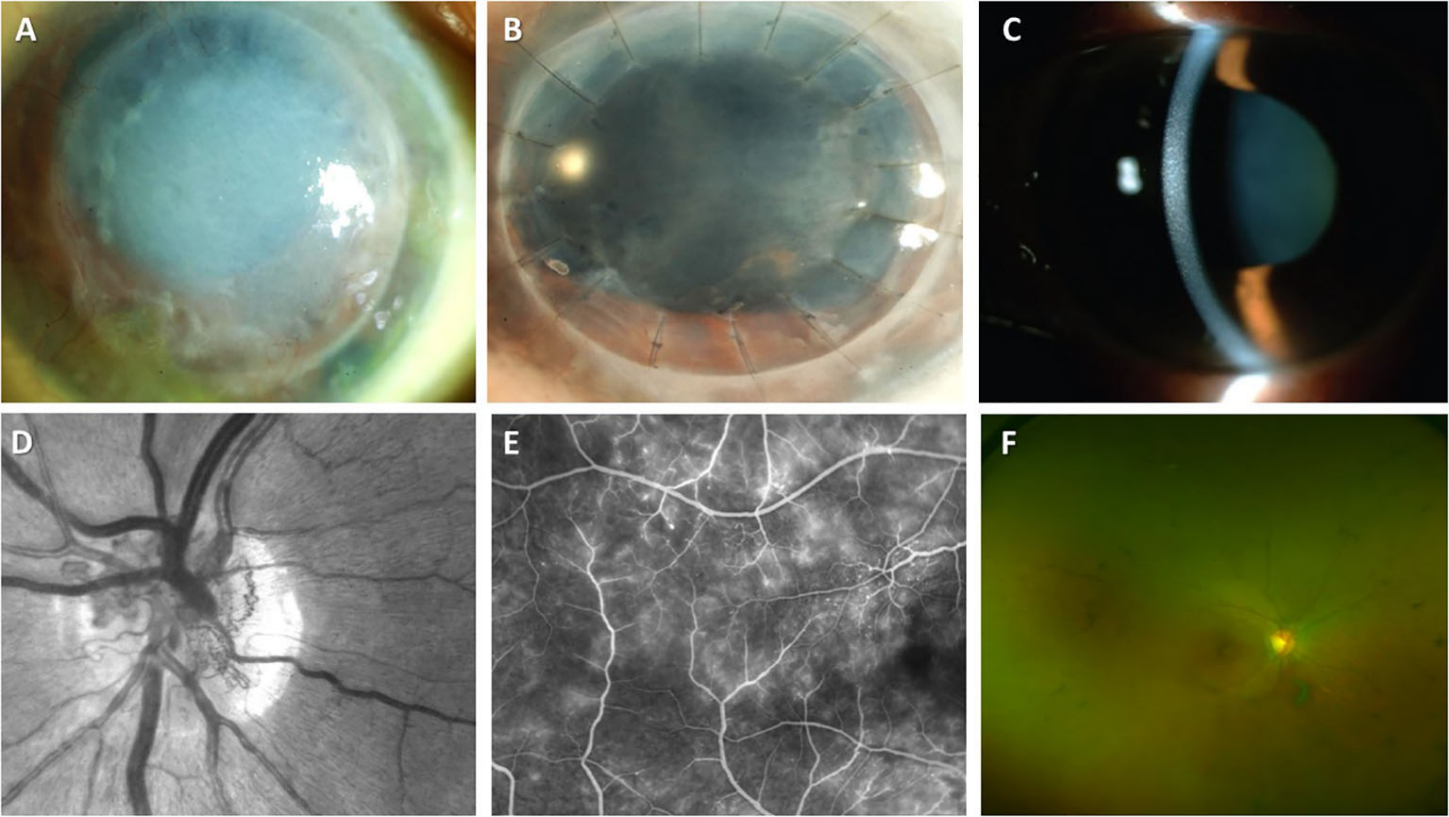
Ocular manifestations of MGCS. **(A-C)** MGCS-associated kerathopathy with visible deposits on slit-lamp examination (**A**, **B** (Image courtesy: Koo, et al., 2011) (182) and **C** (Image courtesy: Kocabeyoglu et al., 2014) (183)); **(D-G)** MGC-associated maculopathy. **(D)** Neovascularization of the disc on the fundal examination (Image courtesy: Gonzales et al., 2021) (184); **(E)** Fluoroscopic angiography demonstrating telangiectasia of vessels and leakage from retinal capillaries (Image courtesy: Gonzales et al., 2021) (184); **(F)** Colored fundus examination showing optic disc pallor, attenuation of retinal vessels, and peripheral pigmentation (Image courtesy: Eton et al., 2020) (185).

Ophthalmologic injury (sometimes referred to as ocular MG) in MGUS involving primarily corneal and retinal layers of the eye is not a common event (**Figure 3**). MG-associated ocular diseases have been also described in MM, B-cell lymphoma, plasmacytoma, Waldenström macroglobulinemia, and CLL. Therefore, the identification of visual acuity impairment with specific corneal lesions should prompt physicians for the hematological evaluation for MGCS (**Table 4**). Conversely, ocular dysfunction may be expected in some patients with MG.

**Table 4:** Literature review on other MGUS-related disorders.

Author/Year	Patients number	MGUS type	Type of involvement	Type of therapy	Outcome	MGUS response
Milman 2015 (186)	5	IgG-κ, IgG-λ	Keratopathy	-Chemotherapy + keratoplasty (1) -BPM + keratoplasty (1) -Keratoplasty (1) -ASHC (1) -CVAF (1)	-Stabilization (3 years) -Recurrence (4 years) -Stabilization (2 years) -Stabilization (7 years) -Recurrence after 1 month	N/A
Branellec 2012 (187)	4	IgG-κ, IgA-λ, IgG-λ	Acquired C1 inhibitor deficiency	Rituximab (4) + C1 inhibitor concentrate (3) + tranexamic acid (1) + IV cyclophosphamide (1) + corticosteroids (1)	Complete (2), partial response (2)	N/A
Cicardi 2003 (188)	23	IgG-λ, IgG-κ, IgM-κ, IgM-λ, IgA-λ	Acquired C1 inhibitor deficiency	-antithrombotic (6) -antifibrinolytic (13) -C1 inhibitor concentrate (12)	-Complete (2), no response (4) -Complete (8), partial (4), none (1) -Complete (9), partial response (3)	N/A
Gobert 2016 (189)	6	IgM-κ, IgM-λ, IgA-κ, IgA-λ, IgG-κ, IgG-λ	Acquired C1 inhibitor deficiency	Rituximab (6)	Complete or partial response (5)	N/A
Bork 2019 (190)	15	IgG, IgM, IgA	Acquired C1 inhibitor deficiency	C1 inhibitor concentrate (15) (+icatibant/rituximab)	Complete response (14)	N/A
Frémeaux-Bacchi 2002 (191)	12	IgM-κ, IgM-λ, IgA-κ, IgA-λ, IgG κ	Acquired C1 inhibitor deficiency	-Danazol + antifibrinolytic/corticosteroid (12) -IV corticosteroids (2) -Chemotherapy (9) -IV Immunoglobulin (12)	-N/A -Complete or partial response (2) -Complete or partial response (7) -No response (12)	MM (1)
Voisin 2011 (192)	14	IgM-κ, IgM-λ, IgG-κ, IgG-λ	Acquired von Willebrand disease	-IVIG (8) -Desmopressin (5) -von Willebrand factor (2)	-Complete response (2) -Complete response (3) -No response (2)	N/A

BPM, bortezomib + prednisone + melphalan; CVAF, corneal vascularization with amniotic membrane graft; MM, multiple myeloma; N/A, not available.

### Paraproteinemic keratopathy

Paraproteinemic keratopathy (also known as keratopathy of monoclonal gammopathy or immunotactoid keratopathy) is caused by bilateral corneal depositions of immunoglobulins mostly of IgG-kappa origin causing distinct opacities and potentially leading to tissue dystrophy with gradual visual acuity loss (183, 193–196). The depositions can be in the form of crystals (called *crystalline keratopathy*) or non-crystalline (peripheral bands/patches/granules, or lattice) (183, 195, 197). In some cases, immunoglobulin-bound copper depositions (reminding Kayser-Fleischer rings of Wilson disease) can also be seen (198, 199). Therapy consisting of MG-specific treatment and reconstructive surgery depends on the severity of ocular involvement. In some cases, the disease might be asymptomatic requiring no intervention but continuous monitoring of MGUS and visual function (197). In more severe cases of ocular involvement and MGUS progression, chemotherapy and/or AHCT aiming at hematological correction can resolve symptoms and stop the disease progression (186, 200). Keratoplasty or corneal transplantation usually carries a short-term benefit for visual repair as high rates of recurrences have been reported (201–204), while topical agents (steroids and tacrolimus) were not shown to be effective (205).

### Maculopathy of monoclonal gammopathy

A much rarer manifestation of ophthalmologic injury in MGUS is maculopathy with the possibility of unilateral or bilateral retinal detachment and subsequent visual loss (206). Clinical presentation may include inflammation of the iris and vitreous body as well as macular detachment (207). Besides immunoglobulin depositions, paraproteins acting as autoantibodies against macula were proposed as potential pathogenesis, however, the exact mechanism of maculopathy in MGUS is unknown (207). Due to a low number of reports, optimal treatment is yet to be discovered. Hematologic targeting (chemotherapy, rituximab, plasmapheresis) may lead to clinical resolution (206, 207), whereas ocular surgery, acetazolamide or eplerenone, and topical agents (glucocorticoids, triamcinolone, dorzolamide, anti-vascular endothelial growth factors) are not always effective with only short-term symptomatic correction of visual function (208, 209).

## Other diseases associated with MGUS

### Acquired C1 inhibitor deficiency

Acquired C1 inhibitor deficiency, also known as acquired angioedema, is a rare disease manifested with recurrent episodes of angioedema of the skin and mucosa of gastrointestinal and upper respiratory tracts (210). Pathogenesis involves autoantibodies against C1 inhibitor, involvement of bradykinin and cytokines, as well as abnormal activity of the classical complement pathway by neoplastic tissue (211). Clinical presentation is identical to hereditary angioedema with the difference that patients with acquired C1 inhibitor deficiency are older (≥40 years of age) with no family history and usually have associated diseases (in 70-85%), including lymphoproliferative disorders, MGUS, and non-Hodgkin lymphoma (NHL), and autoimmune diseases (188, 212). MGUS (without a specific predominant type) has been reported in 30-40% of patients occurring before, at or after the diagnosis of acquired C1 inhibitor deficiency with a low likelihood of progressing into multiple myeloma (188–191). The disease management focuses on acute treatment for angioedema episodes (i.e., C1 inhibitor replacement, fresh frozen plasma, icatibant, or ecallantide), which may prompt intubation in severe cases (188–191, 213). Prophylactic measures for the prevention of episodes include antifibrinolytics (tranexamic acid), corticosteroids, androgens (danazol), or regular use of C1 concentrate (188, 212, 214). Management of associated diseases can also be of significant clinical benefit (191, 212). Rituximab was reported to be effective in two reports, leading to symptomatic relief in eight out of eleven patients in total (187, 206) (**Table 4**).

### Acquired von Willebrand disease

Acquired von Willebrand disease (aVWD) is the less common type of VWD due to an underlying medical disorder affecting von Willebrand Factor (VWF) (215). It corresponds to only 1-5% of all cases with a similar clinical and laboratory presentation also seen in the inherited type of VWD (i.e., spontaneous major and minor bleeding) (215). This disease has been associated with a number of conditions, including cardiovascular disease (216), Wilms tumor in children (217), hypothyroidism (218), autoimmune disorders (219), drug use (220), and hematological malignancies, such as myeloproliferative neoplasms and lymphoproliferative disorders (221), the latter nearly always having an underlying MGUS (192). The prevailing majority have IgG with only a small proportion carrying IgM paraprotein (192, 222). Clinical management of aVWD is similar to the inherited form and the presence of monoclonal gammopathy does not substantially affect the treatment strategy. Correction of bleeding is the main approach, for which desmopressin (DDAVP), factor-replacing therapy with concentrates of VWF/recombinant activated factor VII, as well as antifibrinolytic therapy have been used with varying degrees of success (222–225). IVIG (1g/kg/day for two days with 3-week interval repeats) can also lead to positive outcomes and is most reasonable in cases of related autoimmune diseases (222, 225–227), and a recent systematic literature review found an 85% response rate in patients with MGUS (228) Successful combination of concentrates with IVIG were also reported (229). Limited data also exist regarding the applications of lenalidomide (230), rituximab (231), daratumumab (232) and plasmapheresis (233) (**Table 4**). Clinicians should aim at revealing the underlying condition as its management can alleviate the symptom severity and disease progress.

### Crystal-storing histiocytosis

Crystal-storing histiocytosis (CSH) is a rare condition, in which abnormal immunoglobulins are accumulated in the form of crystals in lysosomes of histiocytes resulting in single-organ or multiorgan damage, involving the kidney, eye, lungs, bone marrow, gastrointestinal tract, or spleen (234). The disease is thus categorized as an Ig deposition disease along with other pathologies caused by MGUS (primarily IgG with kappa light chain) (234–239). Treatment is based on the underlying condition as well as the severity and progression of the clinical picture. Careful monitoring is optimal for limited pathological lesions (234). In more severe circumstances, different chemotherapeutic regimens (bortezomib-based, daratumumab-based, or R-CHOP) led to considerable clinical improvements in case reports (236–239). AHCT was successful in a complex of ocular and periorbital crystal-storing histiocytosis with Fanconi syndrome (200) (**Table 4**).

## Conclusion

The spectrum of various disorders associated with MGUS, in the absence of MM, Waldenström macroglobulinemia, or other lymphoproliferative disorders, describes a new entity of MGCS, the management of which remains a subject of further research and is yet to be determined. Apart from a few diseases, in which specific etiological or pathogenic therapeutic options are known (e.g., anakinra for Schnitzler syndrome or C1 inhibitor concentrate for acquired C1 inhibitor deficiency), treatment of MGCS involves myeloma-targeting agents or immunosuppressive and immunomodulatory medications, depending on the type of the disorder associated with M protein. Future studies are required to deepen our understanding of the pathogenesis of MGCS, which may guide us through the path of finding the optimal treatment for this complex yet intriguing clinical spectrum concerning multiple medical disciplines.

## Author contributions

The manuscript was drafted and edited by AO, who also performed the systematic literature search. The paper was designed and structured by AM. Data extraction was performed by AG. FM, MM, YH, NG, AG, and DK reviewed and edited the article. All authors contributed to the article and approved the submitted version.

## Conflict of interest

The authors declare that the research was conducted in the absence of any commercial or financial relationships that could be construed as a potential conflict of interest.

## Publisher’s note

All claims expressed in this article are solely those of the authors and do not necessarily represent those of their affiliated organizations, or those of the publisher, the editors and the reviewers. Any product that may be evaluated in this article, or claim that may be made by its manufacturer, is not guaranteed or endorsed by the publisher.
