# Supplementary material for: Monoclonal gammopathies of clinical significance (MGCS): In pursuit of optimal treatment
Source: Front Immunol. 2022 Nov 23;13:1045002. doi: 10.3389/fimmu.2022.1045002 (PMC9728929; doi:10.3389/fimmu.2022.1045002)
Supplement: Supplementary file 1 [file DataSheet_1.docx]

Supplementary Material

# Systematic literature search strategy

A systematic literature search through PubMed was conducted from the day of inception up until August, 2022. The two major domains of terms were used: (1) monoclonal gammopathy OR monoclonal gammopathy of uncertain/ unclear/ unknown/ undermined/ underdetermined/ undetermined significance; (2) clinical pathologies (listed in Table 1).

The search was restricted to full-text primary data human studies (case reports, observational studies, clinical trials) published in English, German, French, or Russian languages. Editorials, reviews, commentaries, and viewpoints were not considered eligible unless they contained a primary description of a patient case. Studies were not excluded based on age, sample size or date of publication. References of all initially identified studies were further manually screened for potentially eligibility. Studies met the eligibility criteria if they included primary patient data with detected MG and associated neurological, cutaneous, hematological, or ophthalmological conditions. Articles describing renal pathologies linked to MG (i.e., MGRS) were excluded.

Data extraction was carried out independently by two investigators (AG and AO). The following items were extracted from each publication: study ID, study design, sample size, type of MGCS, type of MGUS or MM, progression to MM, administered therapies, and treatment outcomes. All decisions were made based on a consensus between AG and AO, and conflicts were resolved by a third author (AM).
